# Metrological Comparison of Indirect Calibration Methods for Nanoindentation: A Bootstrap-Based Approach

**DOI:** 10.3390/ma18184382

**Published:** 2025-09-19

**Authors:** Giacomo Maculotti, Lorenzo Giorio, Gianfranco Genta, Maurizio Galetto

**Affiliations:** 1 Department of Management and Production Engineering, Politecnico di Torino, Corso Duca degli Abruzzi 24, 10129 Turin, Italy; gianfranco.genta@polito.it (G.G.), maurizio.galetto@polito.it (M.G.); 2 Department of Applied Science and Technology, Politecnico di Torino, Corso Duca degli Abruzzi 24, 10129 Turin, Italy; lorenzo.giorio@polito.it

**Keywords:** nanoindentation, instrumented indentation test, calibration, uncertainty, bootstrap, frame compliance, indenter calibration

## Abstract

Area shape function and frame compliance are the most critical parameters in nanoindentation, as they control measurement accuracy and represent the largest contributions to measurement uncertainty. Despite the availability of direct calibration methods, indirect calibrations are the most practical and fast. Thus, the indirect calibration methods proposed in ISO 14577-2 are most typically applied in academic and industrial research, as well as in quality controls. Previous research has highlighted some criticalities, but a holistic metrological framework was missing. This work aims to compare the performances of indirect calibration methods for area shape function and frame compliance in the nano-range, considering different alternatives suggested in the standard and most recent literature. The comparison will be based on uncertainty estimation using bootstrap estimation, which will innovatively highlight and introduce the effect of the nanoindentation dataset in the uncertainty estimation. The results show that the optimization of accuracy and uncertainty in mechanical characterization is achieved by indenting pairs of certified reference materials, resulting in a more robust approach to calibration experimental conditions than methods that require a single sample to be indented.

## 1. Introduction

The Instrumented Indentation Test (IIT) in the nano-range is a depth-sensing characterization method [1,2]. Although it was originally conceived as a hardness scale capable of overcoming the limits inherent in the optical resolution of conventional hardness scales, it immediately became highly attractive to practitioners [3].

Nanoindentation enables the mechanical characterization of surface layers [4] in terms of hardness, Young’s modulus estimation, creep, relaxation [5], and toughness [6], while showing a high resolution that can resolve microstructural phases [7,8] and characterize micro- and nano-components [3]. Recently, nanoindentation has found applications in cutting-edge fields such as energy harvesting devices, i.e., micro nano pillars and nanowire characterization [9]; optoelectronics materials [10]; semiconductors, i.e., for characterizing mechanical properties correlated to energy efficiency and passivation [11]; phase transformation [12]; coatings structure [13,14]; and thickness characterization [15]. More recent and advanced applications leverage nanoindentation’s capability of performing high-temperature characterization to identify material properties and temperature-induced phase changes [16], to characterize residual stress [17,18] and dislocation density [19], to study super elasticity, and shape memory in advanced materials [20] and soft materials, e.g., solgels [21], polymers [22,23], and biomaterials, e.g., hydrogels [24,25], artificial tissues [26,27], natural fibres [28], and for early cancer diagnosis [29,30].

**Figure 1:**
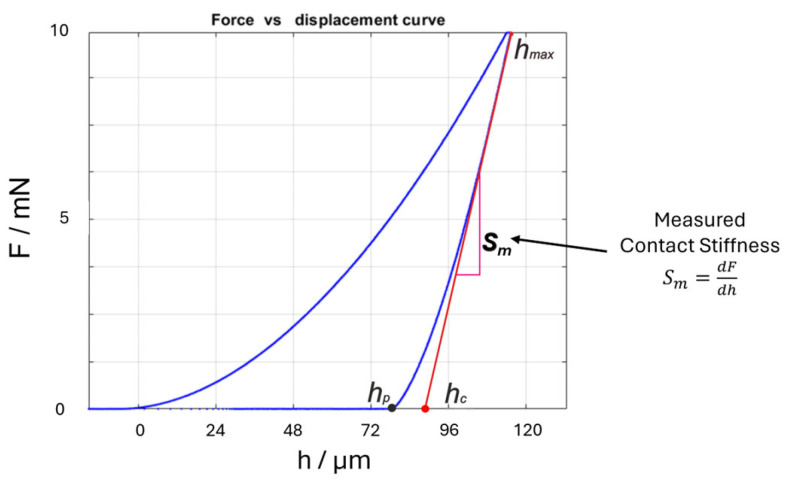
Indentation curve (*IC*): applied force (*F*) as a function of the penetration depth (*h*), with highlighted parts showing the corrected depth (*h_c_*), the residual penetration depth (*h_p_*), and the measured contact stiffness (*S_m_*).

IIT consists of applying a force-controlled indentation cycle of loading, holding, and unloading to a sample by means of an indenter. During the cycle, the applied force (F) and the indenter penetration depth (h) are measured continuously. Figure 1 shows the resulting indentation curve, i.e., F(h).

Mechanical characterization is achieved, as described by ISO 14577-1 [5], in terms of indentation modulus *E_IT_*, i.e., estimating the Young’s modulus, and in terms of indentation hardness *H_IT_*.

(1) $$E_{IT}=\frac{1-\nu_{s}^{2}}{\frac{2\sqrt{A_{p}(h_{c,max})}}{S\sqrt{\pi }}-\frac{1-\nu_{i}^{2}}{E_{i}}}$$

(2) $$H_{IT}=\frac{F}{A_{p}(h_{c,max})}$$

(3) $$h_{c,max}=h_{max}-h_{0}-C_{f}F_{max}-\varepsilon \frac{F_{max}}{\frac{1}{S_{m}}-C_{f}}$$

(4) $$S_{m}={\left.\frac{\partial F}{\partial h}\right|}_{h_{max}}$$

(5) $$C_{tot}=\frac{1}{S_{m}}=\frac{1}{S}+C_{f}$$

where *ν_s_* is the sample’s Poisson’s ratio, *ν_s_* is the indenter’s Poisson’s ratio, and *E_i_* is the indenter’s Young’s modulus. $A_{p}(h_{c,max})$ is the projected contact area at the maximum corrected penetration depth $h_{c,max}$ and *S* is the sample contact stiffness. The corrected contact depth is obtained, as per Equation (3), by correcting the measured penetration for the zero contact point error, $h_{0}$, and for the elastic displacement of the sample, $\varepsilon \frac{F_{max}}{\frac{1}{S_{m}}-C_{f}}$, and of the indentation platform, $C_{f}F_{max}$. In particular, ε is an indenter geometry-related parameter, $S_{m}$ is the measured contact stiffness, defined in Equation (4), and $C_{f}$ is the frame compliance. The sample contact stiffness *S* is estimated by analyzing the unloading curve, modelling the indentation platform–sample system as a set of springs in series according to Equation (5).

It is worth noticing that the key feature that allows the achievement of nano-scale characterization is the relationship between the contact area and the penetration depth, i.e., the area shape function A_p_, typically expressed as a sum of rational monomials as in Equation (6) [2,5].

(6) $$A_{p}\left(h\right)=\sum_{n=0}^{8}a_{n}h_{c}^{2-n}$$

The metrological evaluation of nanoindentation has long been investigated to ensure accurate, traceable, and precise material characterization, with the reporting of measurement uncertainty. ISO 14577-2 describes the calibration of the most relevant influence factors [31]. The calibration of force and displacement sensors allows for traceable measurements. However, further influencing factors are most impactful on uncertainty evaluation [32]. On the one hand, the surface integrity is liable for dominating measurement uncertainty by largely increasing the reproducibility [5]. In stable conditions, the most relevant contributors are the area shape function parameters *a_n_*, the frame compliance *C_f_*, the contact stiffness *S_m_*, and the first contact point *h_0_* [32].

The evaluation of *S_m_* and *h*_0_ significantly impacts both accuracy and precision, depending on the chosen algorithm for their evaluation [32,33,34]. The literature has shown that among ISO standard methods, the Power Law (PL) method allows for the most accurate and precise material mechanical characterization [32]. In particular, the loading and unloading curves can be modelled as per Equation (7a,b) as follows:

(7a) $$h=\alpha_{l}{\left(h-h_{0}\right)}^{m_{l}}$$

(7b) $$h=\alpha_{u}{\left(h-h_{p}\right)}^{m_{u}}$$

where *α* and *m* are material and indenter geometry related parameters, the subscripts *l* and *u* stand for loading and unloading, respectively, and *α* and *m*, $h_{0}$ and $h_{p}$ are obtained by the non-linear least square fitting of the relevant portion of the indentation curve [5]. Further approaches have been developed, and direct derivative evaluation methods have been proven to provide an estimation of the *S_m_* that is consistent with the ISO 14577-1 approaches, but with a substantially improved uncertainty [35,36].

Conversely, the area shape function parameters and the frame compliance are calibrated; thus, they not only significantly affect the accuracy and uncertainty but also contribute to the traceability of the method. When discussing the calibration of area shape function and frame compliance, it is first relevant to recall that the measurand are, respectively, the geometrical description of the projected area as a function of the penetration depth, i.e., as a function of the indenter height—for pyramidal indenters—or the radius parallel to the loading direction—for spherical indenters—and the compliance to be ascribed to the machine inducing elastic deformation at the measured penetration depth. The area shape function and the frame compliance, as explicated in Equation (3) and in Equation (5), are necessary to account for the fact that the system is not ideal. In fact, both manufacturing errors and wear, generated by mechanical friction and impurity attachment, e.g., by heat, chemical affinity, make the indenter deviate from the nominal geometry, i.e., an ideally sharp pyramid or a perfect sphere. Such deviations introduce severe bias, which, if not corrected, is most severe at the nano and low micro scale. The frame compliance, on the other hand, accounts for the fact that the system is not infinitely stiff. Thus, the system, due to reaction forces, is subjected to elastic deformations that add up to the measured penetration depth, becoming most significant for higher loads.

### 1.1. Calibration Methods for Area Shape Function and Frame Compliance

The literature describes several calibration methods for area shape function parameters $a_{n}$ and the frame compliance *C_f_*. These calibration methods can be distinguished into direct and indirect approaches.

Direct calibration methods require directly estimating the measurand by a measuring instrument that is traceable. For the area shape function, this is typically achieved using surface topography measuring instruments, which are most suitable for calibrating indenters used for micro- and macro-scale IIT. Several examples can be found in the literature. Typically, national metrological institutes rely on confocal microscopes or coherence scanning interferometers [37], and measurement uncertainty propagation may require the non-trivial linear algebra modelling of geometrical entities [38]. Conversely, at the nano-scale, atomic force microscopy (AFM) is required [39,40,41]. Such an alternative is particularly challenging for extended measurements in both the z-height range and x-y range, possibly requiring stitching, and for deformation and cross-talks of scanning axes [41,42]. The direct calibration of frame compliance can be achieved by compensation balances [43] or springs of known stiffness whose the design and material depend on the force scale, e.g., silicon for the nano-scale [44], or more robust alternatives for the macro-scale [45].

Indirect calibration methods are based on statistical analysis using the fundamental equations of nanoindentation of data collected by indenting one or more reference materials at different loads. Indirect calibration methods are most commonly used thanks to their great practicality, speed, and simplicity compared with direct calibration methods. On the other hand, the uncertainty of calibrated parameters results in larger contributions. Several indirect calibration methods have been proposed; some are suitable only for the macro-scale, some for the nano-scale, and others can be applied to both.

ISO 14577-2 considers some calibration methods, which are typically applied in research laboratories and implemented in testing machine software by manufacturers [31]. Table 1 summarizes the methods currently in use according to the standard. As Table 1 shows, ISO 14577-2 only considers indirect calibration methods for *C_f_*, while direct calibration is also suggested for the area shape function. As mentioned, methods requiring direct calibration of the area shape function, i.e., method #1 [46] and #3 [47], are rarely of interest for industrial and academic applied research laboratories, which, thus, mostly resort to method #2 [48] and #4 [49] for Berkovich and Vickers indenters. Indirect calibration methods require collecting sets of at least 10 indentations at different loads on one or two reference materials. These materials must be suitable to enhance measurand effects, that is, a quite stiff material, e.g., tungsten (W), to maximize the contribution of machine elastic deformation to penetration depth for the frame compliance calibration, and a more elastic material, e.g., Al or SiO_2_ (fused silica “FS”), to allow large penetration depths at small load to increase the accuracy of the area calibration. Method #5 is specific to spherical indenters, while method #6 has been introduced in the latest draft version of the standard for the micro- and macro-ranges.

Some calibration methods might require the use of a certified reference material (CRM) calibrated in terms of $E_{r}$, i.e., the reduced modulus defined as

(8) $$E_{r}={\left(\frac{1-\nu_{s}}{E_{IT}}+\frac{1-\nu_{i}}{E_{i}}\right)}^{-1}$$

while others, i.e., method #2 and #6, allow for autocalibration.

Following the previous brief discussion, the next sections discuss the most commonly resorted to standard calibration method for the nano-range. The discussion is limited to the calibration of a Berkovich indenter. First, a brief description of standard methods is provided along with a discussion of some procedural limitations highlighted in the literature, which prompted the development of a further alternative.

#### 1.1.1. ISO 14577-2 Method #2 (M2)

This method is the most commonly applied in research laboratories and implemented in the software of testing machines. It requires collecting *J* ≥ 10 indentations at *I* force levels. Since the calibration method aims to estimate both frame compliance and area shape function parameters, a relatively elastic material should be used, such as SiO_2_ or Al. The latter, however, is greatly affected by pile-up and may not be an actual ideal choice.

**Figure 2:**
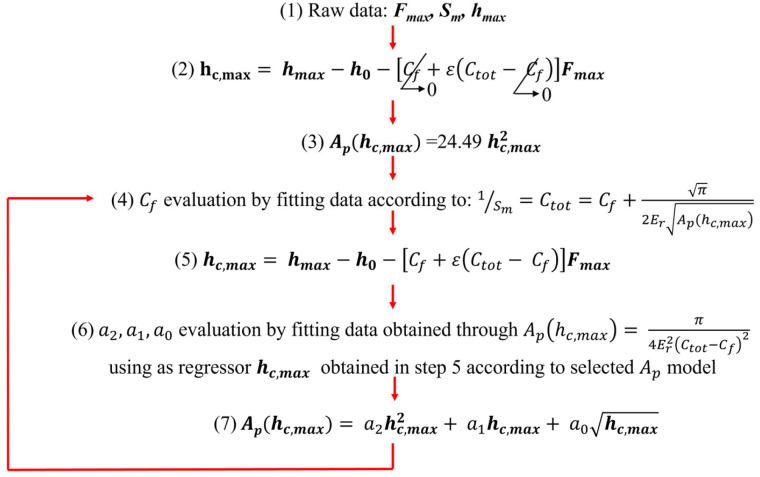
The iterative process for the application of the indirect calibration of area shape function parameters and frame compliance as per ISO 14577-2 methods #2 and #4. The M4 requires carrying out step 4 on the stiffer material (W) and step 6 on the more elastic material (SiO_2_). Step 7 shows one of the several possible models for the *A_p_*. Adapted from [50].

The method, as defined in ISO 14577-2, is an autocalibration method, as no external reference is required. Once the *IJ* indentations have been collected, the maximum force *F_max_*, maximum penetration depth *h_max,_* and measured contact stiffness *S_m_* are evaluated (step 1 of Figure 2), and the problem is initialized assuming an ideal indentation testing machine, i.e., having infinite stiffness (*C_f_* = 0), and ideal indenter geometry ($A_{p}=24.49h_{max}^{2}$), i.e., respectively, step 2 and 3 of Figure 2. Then, a linear regression is performed based on Equation (5) to obtain a first estimate of the frame compliance. Here, it is worth noticing that if the autocalibration approach is applied, i.e., *E_r_* is not known and calibrated, the regression of Step 4 also estimates the ratio $\beta =\frac{\sqrt{\pi }}{2E_{r}}$ which is then used in the iteration cycle. The first attempt value of *C_f_* allows the estimation of the corrected contact depth (step 5), which is used as a regressor term for the known *A_p_*—estimated (in step 6) by the fundamental relationship between *E_r_* (obtained as $\beta^{2}$), *S,* and *A_p_*—for estimating regression parameters of the area shape function (in step 6). Then, from the estimation of the projected contact area (in step 7), the process is iterated until convergence is achieved.

#### 1.1.2. ISO 14577-2 Method #4 (M4)

This approach is a variation on M2. It requires performing two sets of *J* ≥ 10 indentations at *I* force levels on two CRMs (see Table 1). The method then proceeds by applying the iterative procedure outlined in Figure 2 and described in the previous Section 1.1.1. The differences lie in the fact that the steps aimed at evaluating the *C_f_*, i.e., steps 3, 4, and 7, are performed on data collected on the stiffer sample, e.g., W; conversely, steps that allow the evaluation of the area shape function parameters, i.e., steps 5, and 6, are performed on data collected on the more elastic sample, e.g., SiO_2_. Also, since samples are calibrated CRM, *E_r_* is known, so the regression in step 4 shall not estimate the coefficient of the regressor term $\frac{1}{\sqrt{A_{p}(h_{c,max})}}$.

#### 1.1.3. Remarks on ISO 14577-2 M2 and M4

The literature highlights the use of known *E_r_* for the samples, i.e., M4 converges much faster than M2 [31,48,49]. Also, from a statistical perspective, the regressions implemented in steps 4 and 6 are constrained linear least squares problems. Specifically, step 4 requires estimating *C_f_* such that it satisfies $C_{f}>0$, to reflect the physical meaning of the compliance of a mechanical system. Conversely, the constraint for the slope of the model, i.e., $\beta =\frac{\sqrt{\pi }}{2E_{r}}$, depends on the applied method. For M2, since it is an autocalibration, it is only required that β>0. On the other hand, for M4, since calibrated CRMs are used, the actual regressor term can be considered $\frac{\beta }{\sqrt{A_{p}(h_{c,max})}}$. This implies that the slope of the estimated model has to be equal to 1. This constraint for practical implementation, due to numerical solution, can be constrained to $\left[1-\eta ;1+\eta \right]$, where η is a positive, small enough real value, e.g., 0.005. Similarly, constraints are required for the regression of step 6. In particular, $a_{2}>0$, for it is linked to the geometry of the indenter. More stringent requirements can be enforced, e.g., $a_{2}=24.49$ for a modified Berkovich indenter, but the literature has shown that this yields a very large uncertainty on the *A_p_* [51]. Furthermore, it is critical to constrain the linear least squares problem such that $A_{p}>0$. This is a computationally non-trivial requirement. In fact, most relevantly for M4, some estimated parameter sets may lead, for very small $h_{c,max},$ to negative areas. This is not only physically impossible in practice but also leads to numerical issues in the iterative procedure when estimating $A_{p}$ on the stiffer material (in step 7) to move to the next iteration cycle.

Lastly, it is worth noting that if the indenter area shape function is independently and directly calibrated, method #1 and method #3 can be applied. In particular, the procedure of M1 requires applying steps from step 4 onwards of the iterative approach until convergence is achieved, also estimating β with similar constraints as for M2. Conversely, M3 allows a simpler model, as for M4 since it requires a calibrated *E_r_*, as reported in Table 1.

#### 1.1.4. Single-Step Calibration Method (ODR)

From a mathematical perspective, the iterative approach implemented by ISO 14577-2 M2 and M4 ultimately attempts to solve a regression for parameters *C_f_* and *a_n_*. Accordingly, the literature more recently has proposed a more formal rewriting of the approach [50]. The approach requires collecting *J* ≥ 10 indentations at *I* force levels on two CRMs, and solving the multivariate non-linear regression as follows:

(9) $$\left\{\begin{array}{c} \frac{\pi }{4E_{r}^{2}}={\left(\frac{1}{S_{m}}-\;C_{f}\right)}^{2}\left\{a_{2}{\left[h_{max}-h_{0}-\left[C_{f}+\varepsilon \left(\frac{1}{S_{m}}-\;C_{f}\right)\right]F_{max}\right]}^{2}+\;a_{1}\left[h_{max}-h_{0}-\left[C_{f}+\varepsilon \left(\frac{1}{S_{m}}-\;C_{f}\right)\right]F_{max}\right]+\;a_{0}{\left[h_{max}-h_{0}-\left[C_{f}+\varepsilon \left(\frac{1}{S_{m}}-\;C_{f}\right)\right]F_{max}\right]}^{1/2}\right\}\\ H_{IT}=\;\frac{F_{max}}{a_{2}{\left[h_{m}-h_{0}-\left[C_{f}+\varepsilon \left(\frac{1}{S_{m}}-\;C_{f}\right)\right]F_{max}\right]}^{2}+\;a_{1}\left[h_{m}-h_{0}-\left[C_{f}+\varepsilon \left(\frac{1}{S_{m}}-\;C_{f}\right)\right]F_{max}\right]+\;a_{0}{\left[h_{max}-h_{0}-\left[C_{f}+\varepsilon \left(\frac{1}{S_{m}}-\;C_{f}\right)\right]F_{max}\right]}^{1/2}} \end{array}\right..$$

The solution to the non-linear regression problem is best addressed by orthogonal distance regression (ODR). ODR accounts for errors in regression variables due to uncertainty in measuring the penetration depth and applied force, as well as in estimating the first contact point and measured contact stiffness. Since the estimation of the parameters is achieved by solving the regression, this approach can be referred to as a single-step method, as opposed to the standard iterative approach.

The model of Equation (9) is written considering a particular choice of the area shape function; however, other choices are equally applicable, as discussed in Section 1. In particular, Equation (9) is written for a Berkovich indenter assuming a specific truncation of the area shape function reported in Equation (6). By changing the area shape function, it is possible to cater for other approximation models, or for other indenter geometries, e.g., Vickers. Applications of the ODR single-step method to indenter geometries different from Berkovich indenters and to scales larger than nanoindentation are still unreported.

Most relevantly, the ODR single-step method requires the calibration of CRMs in terms of *E_r_* and *H_IT_*. The former is conventionally available. The second is more challenging and less trivial, since the indentation hardness is a parameter highly sensitive to both indentation size effect and pile-up and sink-in. However, under the assumption of the ISO 14577-2, the most commonly used CRMs, i.e., SiO_2_ and W, are purposely chosen to minimize such effects in indirect calibration methods. From a practical perspective, the calibration of *H_IT_* can be obtained using a nanoindentation machine whose *C_f_* and *A_p_* parameters were directly and independently calibrated.

The regression is still subject to constraints, requiring $C_{f}>0$ and $a_{2}>0$. With a suitable large range of penetration depths, conveniently achieved by the selection of CRMs, the constraint on $A_{p}>0$ is no longer necessary, because it was induced by the iterative process attempting to achieve an independent solution of a coupled problem. Lastly, it is worth highlighting that the ODR solves a total least squares problem weighted for the uncertainty of both regressor and dependent variables, i.e., *E_r_* and *H_IT_* [50,52].

### 1.2. Criticalities of Indirect Calibration Methods

From the previously presented literature review, it is clear that iterative approaches lack a formal statistical definition, which is overcome by the ODR single-step approach [50]. An additional discussion can be prompted by the suggestion provided by the ISO 14577-2, which suggests, for M4, to perform measurements on the stiffer material at much higher forces, i.e., considering a (1–100) mN range for the elastic material and a (100–200) mN range for the stiffer material. This suggestion, although sensitive from a practical perspective, as it allows larger indentations to be performed, introduces a decoupling in the evaluation range of frame compliance and area shape function parameters. The literature has more recently shown that frame compliance is non-linear; thus, such decoupling might be liable to bias the estimation of calibrated parameters. However, investigations of such decoupled force ranges on parameters’ calibration are not available, to the authors’ best knowledge. Moreover, the ISO 14577-2 states that M4 allows for estimating a non-constant frame compliance. However, since the *C_f_* is estimated as the intercept of a linear model, which is evaluated on a certain force range, the method implies that a constant *C_f_* is actually estimated on the considered force range. A strong piecewise linear approximation of the non-linear behaviour of *C_f_* could be obtained by applying the M4 method on several force ranges.

Furthermore, from an uncertainty evaluation perspective, iterative approaches hinder the application of closed-form estimations, as the law of propagation of uncertainty, because each iteration relies on statistically estimated parameters that have their own variability [50,51,53]. This requires the application of simulative approaches for uncertainty evaluation. The literature has attempted comparisons based on the Monte Carlo method, for which it has been proven more recently that a proper set up is highly critical due to the correlation of input quantities, i.e., the applied force and the penetration depth [54]. Previous results, based on the Monte Carlo method, showed a substantial impact of the range of *I* maximum forces on the calibrated parameters for the ISO 14577-2 methods [50,51,53]. Moreover, M2 being presented as an autocalibration approach, despite being highly convenient and cheaper, might result in increased measurement uncertainty. More recently, an improved bootstrap-based approach for uncertainty evaluation was proposed. This is particularly convenient for practitioners, for it does not require a strong distributional hypothesis to be performed on input quantities. Conversely, it highlighted a potential severe underestimation of the measurement uncertainty of calibrated parameters when indirect calibrations are performed only on a single set of *IJ* indentations [54]. However, the previously highlighted sensitivity for the number and range of *I* loads was no longer investigated with the most appropriate bootstrap-based uncertainty evaluation. Similarly, no report of such effects is available for the ODR single-step method.

### 1.3. Scope of the Work

This work focuses on indirect calibration methods for area shape function and frame compliance for nanoindentation. With reference to the criticalities highlighted in Section 1.2, this paper aims to describe the performances of the most commonly used methods suggested in the ISO 14577-2 and of the main alternatives that have been proposed within a metrological framework. This is achieved by comparing the parameters calibrated using diverse methods and assessing their effect on mechanical characterization results, while considering calibration uncertainty. Furthermore, leveraging previous partial investigations in the literature, a structured sensitivity analysis on the main factors liable for affecting the calibration results of such methods, dependent on particular user’s choices, will be investigated. Ultimately, this paper aims to provide practical guidelines for the optimal use and implementation of standard calibration methods, while highlighting the potential of other approaches, with the goal of enabling practitioners to minimize the bias and uncertainty of characterization by nanoindentation.

The rest of the paper is structured as follows. Section 2 describes the experiments implemented for the application of selected calibration methods and the approach for the uncertainty evaluation of the results. Section 3 presents the results that are discussed in Section 4. Finally, Section 5 will conclude on the findings.

## 2. Materials and Methods

This paper aims to compare the sensitivity of the ISO 14577-2 M2 and M4 and of ODR calibration methods to the force range considered for calibration. Furthermore, the effect on the accuracy and precision of autocalibration will be assessed. The comparison will be based on experiments described in Section 2.1 and will assess the effect of different calibration methods on the average and measurement uncertainty of calibrated parameters, namely the frame compliance *C_f_* and the area shape function parameters *a_n_*, as well as on mechanical characterization in terms of the indentation modulus *E_IT_*. The comparison will exploit hypothesis tests based on the t-Student distribution and the uncertainty evaluation method described in Section 2.2.

### 2.1. Experiment Set Up

Indentations were performed by a state-of-the-art Anton Paar (Neuchatel, CH) NHT^3^ indentation platform hosted in the metrological room of the Mind4Lab@DIGEP. The indentation instrument was equipped with a Berkovich indenter. The indentation platform features calibrated force transducers with an accuracy of ±1% of the reading and a resolution of 1 nN, and a capacitive displacement sensor with an accuracy of ±0.05% of the reading and a resolution of 0.04 nm. Indentations are performed on a CRM calibrated by NPL in terms of Young’s modulus and Poisson’s ratio by the pulse–echo method. Table 2 summarizes the considered maximum loads and the calibrated values for each CRM. For each load, *J* = 15 replications were performed; each indentation consisted of a force-controlled cycle with a loading to the maximum force and a complete unloading both for 30 s, and a holding time of 10 s (for the SiO_2_) and of 60 s (for the W). Measurements are performed with an acquisition frequency of 10 Hz. As can be appreciated from Table 2, a slight decoupling in terms of the investigated force ranges is necessary to avoid the indentation size effect and pile-up to bias measurement results on W. The collected data were used to calibrate the frame compliance and the area shape function of the indenter according to the calibration methods listed in Table 3.

### 2.2. Uncertainty Evaluation

The uncertainty of the calibrated parameters is evaluated using a bootstrap approach proposed elsewhere and summarized here. As briefly discussed in Section 1.2, bootstrapping does not require modelling the correlation between the input quantities, i.e., the force and the penetration depth. This greatly simplifies the approach compared with parametric simulative approaches, such as Monte Carlo, which, if applied in a way that neglects the correlation results in an excessive overestimation of measurement uncertainty [54].

The bootstrapping requires for each set of *J* replicated indentations (at a given i-th load) to resample the experimental curve, which contains *B* points. The resampling is performed by randomly extracting time-subsequent pairs of $\left\{F,h\right\}$ from the *J* curves. Resampling $\left\{F,h\right\}$ pairs allow us to inherently cater for the correlation *F(h)*. Each calibration requires a set of *IJ* curves. By applying the resampling to each of the *I* indentation sets collected at different loads, it is possible to simulate a new calibration dataset, i.e., a bootstrap sample. The number of simulated calibration datasets is $K(<K^{B-1})$. Accordingly, it is possible to perform *K* calibrations of *C_f_* and *a_n_*. In this work, *K* = 11,000 simulated calibration datasets were generated.

If we let $\hat{x}$ be any of the calibrated parameters, it is possible to compute the grand average representing the calibrated value as follows:

(10) $$x_{cal}=\bar{\hat{x}}=\frac{\sum_{w=1}^{K}{\hat{x}}_{w}}{K},$$

and—with ANOVA modelling—considering the variability due to the different calibration datasets (variance between $s_{B}^{2}$) and the variability due to the regression (variance within $s_{W}^{2}$) it is possible to firstly assess the significance of the calibration dataset on the calibrated parameter, and secondly to obtain the total variance of the calibrated parameter $s_{TOT}^{2}$, by composing the variance between and the variance within, as per Equation (13). In particular, the variance within is obtained as the average of the variance of the regression estimated variability, i.e., the square of the standard error. The last standard error from the iterative procedures can be used for computations. From the total variance, it is possible to estimate the expanded uncertainty of the calibrated parameter, as in Equation (14), which approximates the coverage factor with the value 2, considering the *KIJ-1* degrees of freedom.

(11) $$s_{B}^{2}=IJ\cdot \frac{\sum_{w=1}^{K}{\left({\hat{x}}_{w}-x_{cal}\right)}^{2}}{K-1}$$

(12) $$s_{W}^{2}=\frac{\sum_{w=1}^{K}{SE}_{w}^{2}(\hat{x})}{K}$$

(13) $$s_{TOT}^{2}=u^{2}\left(\hat{x}\right)=\frac{s_{B}^{2}\cdot \left(K-1\right)+s_{W}^{2}\cdot K(IJ-1)}{KIJ-1}$$

(14) $$U\left(\hat{x}\right)=2s_{TOT}$$

Furthermore, the effect of the possible differences on calibrated parameters on mechanical characterization is assessed. This is investigated for the main mechanical characteristics, i.e., *E_IT_*. Additionally, the expanded uncertainty of *E_IT_*, i.e., $U\left(E_{IT}\right)=2u\left(E_{IT}\right)$, is evaluated by applying the law of propagation of uncertainty to their definition reported in Equation (1) [32,54,55], assuming a coverage factor *k* = 2, corresponding to a confidence level of 95%. The average value of *E_IT_* is directly evaluated from experimental data by applying Equation (1) and considering the average of the results.

### 2.3. Statistical Evaluation of Results

The relevance of the calibration dataset and the effect of different calibration methods will be discussed in terms of the following:

- The distribution shape of the calibrated value ${\hat{x}}_{w}$.
- The statistical relevance of the effect of calibration datasets by performing a hypothesis test based on the F-Fisher distribution, with the null hypothesis $H_{0}:\;\frac{s_{B}^{2}}{s_{W}^{2}}\sim F_{K-1,K(IJ-1)}$, where the degrees of freedom of the numerator are *K* − 1 and of the denominator are *K*(*IJ* − 1).
- The statistical difference in calibrated values by performing a hypothesis test based on the t-Student distribution, with the null hypothesis $H_{0}:\;\frac{{\hat{x}}_{C1}-{\hat{x}}_{C2}}{\sqrt{u^{2}\left({\hat{x}}_{C1}\right)+u^{2}\left({\hat{x}}_{C2}\right)}}\sim t_{\upsilon_{pooled}}$, where ${\hat{x}}_{C1}$ and ${\hat{x}}_{C2}$ are the calibrated parameters from any two methods from Table 3, and $\upsilon_{pooled}$ are the pooled degrees of freedom of the difference ${\hat{x}}_{C1}-{\hat{x}}_{C2}$ obtained by the Welch–Satterthwaite formula [55].
- The statistical difference in mechanical characterization results by performing a hypothesis test based on the t-Student distribution, with the null hypothesis $H_{0}:\;\frac{{E_{IT}}_{C1}-{E_{IT}}_{C2}}{\sqrt{u^{2}\left({E_{IT}}_{C1}\right)+u^{2}\left({E_{IT}}_{C2}\right)}}\sim t_{\upsilon_{pooled}}$.
- Relative accuracy: $\frac{{E_{IT}}_{Ci}-{E_{IT}}_{ref}}{{E_{IT}}_{ref}}$, where ${E_{IT}}_{Ci}$ represents the mechanical characterization from any of the considered calibration methods (in this case, *i* ranges from 1 to 10), and the ${E_{IT}}_{ref}$ is the reference value reported on the calibration certificate by an independent characterization method.
- Relative uncertainty: $\frac{U\left({E_{IT}}_{Ci}\right)}{{E_{IT}}_{ref}}$.

## 3. Results

**Figure 3:**
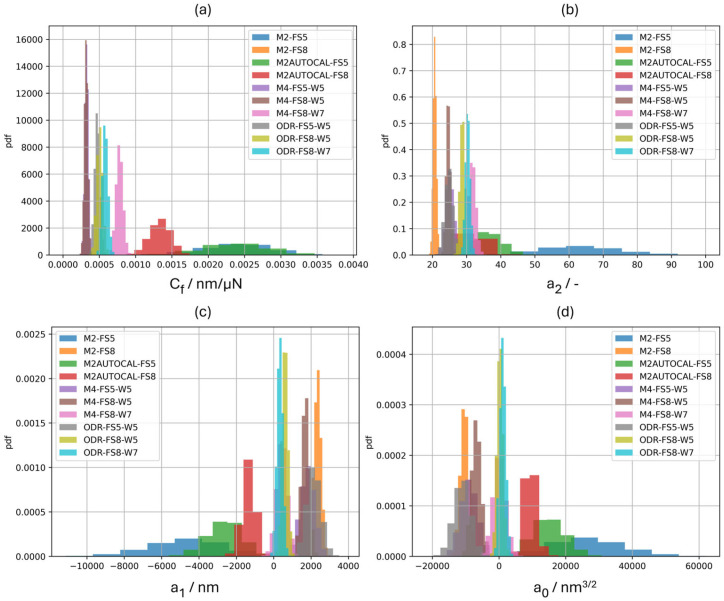
Empirical probability density function (pdf) of the 10,000 calibrated parameters for the 10 calibration methods. (**a**) Frame compliance (*C_f_*): notice the calibration method dependency of the dispersion, and the difference in the average estimates due to different calibration methods. (**b**) *a*_2_: theoretical value is 24.49; notice the significant dependence of both average and dispersion on the calibration method for all area shape function parameters, i.e., also for (**c**) *a*_1_ and (**d**) *a*_0_. Due to the scale, the histogram of *C_f_* for the calibration method M2-FS8 is not reported. Appendix A reports individual histograms for *C_f_* (see Figure A1).

The ten different calibration methods were applied according to the experimental plan outlined in Section 2.1. The bootstrap approach was applied to elicit the uncertainty evaluation of the calibrated parameters, and 10,000 bootstrap samples were generated. Figure 3 shows the histogram of the calibrated parameters (*C_f_)* and the area shape function parameters (*a_n_*).

According to Equations (11) and (12), the variance of the mean calibrated parameters (variance between bootstrap sample calibration) and the variance due to regression (variance within bootstrap sample calibration) were estimated and combined to evaluate the total variance of the calibrated parameters.

Table 4 reports, for each calibration method and for each calibrated parameter, whether the contribution to the total variance of the calibrated parameter due to the calibration dataset (here simulated by bootstrapping), i.e., the variance between, is statistically significant with a risk of error of 5%. Also, the table reports the relative contribution of the bootstrapping to the sum of squares (*SS*), i.e., the product of the variance and the relevant degrees of freedom.

**Figure 4:**
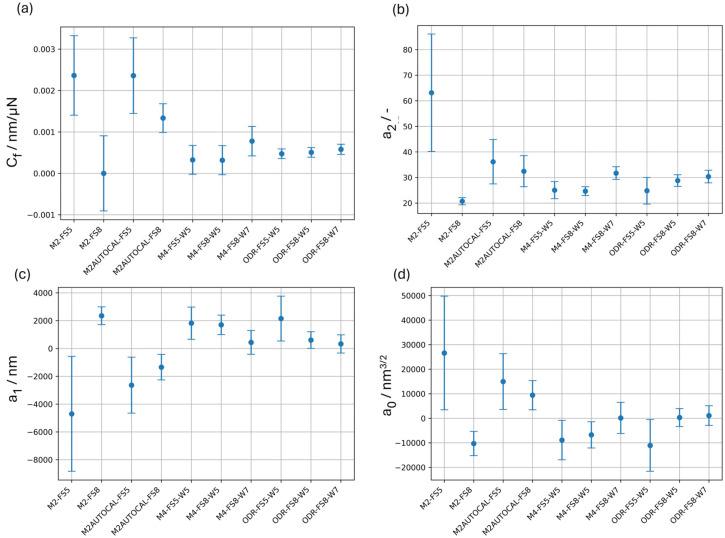
Error bar plots of the calibrated parameters as a function of the calibration method. (**a**) frame compliance, (**b**) *a*_2_, (**c**) *a*_1_, and (**d**) *a*_0_. Error bars represent the expanded uncertainty with a coverage factor of 2 (confidence level of 95%).

Confidence intervals with a confidence level of 95%, i.e., with a coverage factor of 2, are reported for the calibrated parameters in Figure 4. Confidence intervals are evaluated from the expanded uncertainty as per Equation (14), and the error bar plots allow for a comparison of the effect of the calibration method.

**Figure 5:**
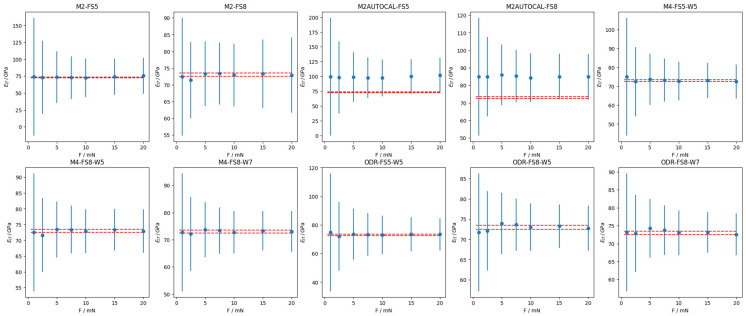
SiO_2_ *E_IT_* for the different calibration methods. *E_IT_* as a function of maximum characterization force. Error bars represent expanded uncertainty (coverage factor of 2). Red dashed lines are the confidence interval for the independently calibrated value of the Young’s modulus of the sample.

**Figure 6:**
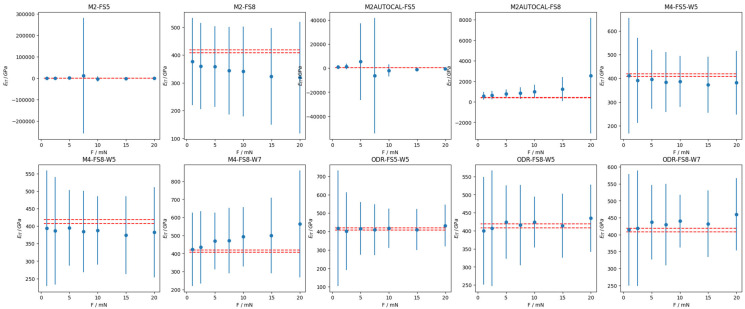
W *E_IT_* for the different calibration methods. *E_IT_* as a function of maximum characterization force. Error bars represent expanded uncertainty (coverage factor of 2). Red dashed lines are the confidence interval for the independently calibrated value of the Young’s modulus of the sample.

Finally, the mechanical characterization of the calibrated samples (see Table 2) has been evaluated in terms of *E_IT_*. Figure 5 and Figure 6, respectively, show the validation of the calibration methods on the SiO_2_ and the W CRM, where error bars represent the expanded uncertainty evaluated according to the law of propagation of uncertainty with a coverage factor of 2. Since average values are computed from experimental data, changing only the calibrated values of the area shape function parameters and the frame compliance, any change in the average and the expanded uncertainty in the panels of Figure 5 and Figure 6 must be ascribed to the calibration methods.

**Figure 7:**
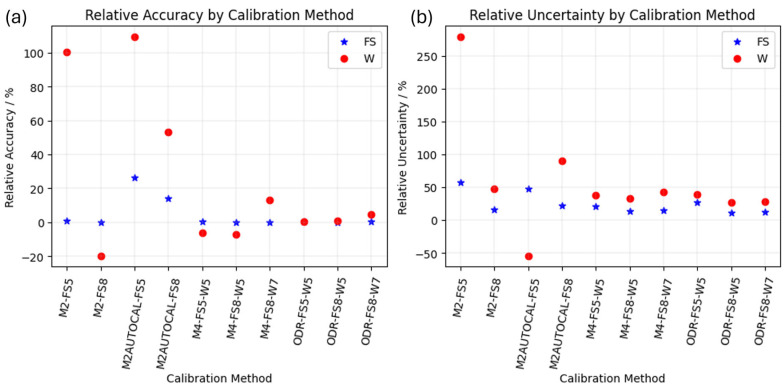
(**a**) Relative accuracy and (**b**) relative uncertainty for the *E_IT_* of SiO_2_ (FS blue star) and W (red circle). Improved visualization with large values excluded is available in Figure A2.

The relative accuracy and relative expanded uncertainty for the obtained *E_IT_* of the two CRMs are reported in Figure 7a,b, respectively.

## 4. Discussion

As can be seen from Figure 3, the different calibration methods impact the average and the dispersion of the calibrated parameters. In particular, methods based on only one CRM, i.e., M2 and M2AUTOCAL, provide a much larger dispersion of and average *C_f_* and *a*_2_.

The ANOVA, as highlighted in Table 4, shows that for all parameters, the use of a single indentation set (i.e., a group of *J* replications performed at *I* loads) is liable to severely underestimating the measurement uncertainty of the calibration parameters. The only exception is represented by the method M4 of ISO 14577-2, when the highest loads are considered for W, i.e., M4-FS5-W5 and M4-FS8-W5. The ANOVA result highlights the need for multiple calibration datasets to be collected to allow a representative estimation of the measurement uncertainty. This can be effectively achieved by bootstrapping to relieve the experimental cost.

Once the total variance is estimated and the expanded uncertainty of the calibrated parameters is evaluated, it is possible to perform hypothesis tests to quantify if the differences are statistically significant. This can be obtained graphically by the error bars in Figure 4. As can be appreciated, methods based on a single CRM, i.e., M2 and M2AUTOCAL, provide a systematically different estimation of all calibrated parameters.

Such results reflect on the evaluation of the mechanical characterization in terms of indentation modulus *E_IT_*. On the one hand, as can be appreciated in Figure 5 and Figure 7a, the evaluation of SiO_2_, i.e., fused silica (FS), is on average accurate, apart for the exception of the M2AUTOCAL method, which results in a severe, statistically significant bias of more than 15%. On the other hand, as shown in Figure 6 and Figure 7a, methods based on a single CMR have very limited applicability to different materials, yielding severe bias (larger than 50%) and considerably large relative uncertainties.

A closer insight into the methods based on one CRM reveals that autocalibration results in a larger relative uncertainty and poorer accuracy, as highlighted by the comparison of results from M2 and M2AUTOCAL in Figure 7. Furthermore, it is immediately apparent that such methods are sensitive to the range of considered forces. Specifically, the adoption of a larger force range (FS8, which adds larger forces, i.e., 15 mN and 20 mN) on a more elastic sample (SiO_2_) is liable to superposing the non-linearity of the system compliance with a contribution due to the elastic deformation that becomes increasingly negligible due to the larger overall penetration depths. This results in a systematic trend in the mechanical characterization results of the stiffer sample, i.e., W, as shown in Figure 6. This degenerates for the M2-FS8. In this case, the estimated frame compliance is 0 nm/μN, due to the mathematical constraints. This is counterbalanced by a very large standard error, which motivates why the ANOVA cannot highlight systematic relevance for the indentation calibration dataset obtained by bootstrap. Such a frame compliance estimation of 0 nm/μN is due to the constraint of the regression requiring a positive *C_f_*, as described in Section 1.1.3. Apparently, the M2-FS8 would have resulted in a negative frame compliance, which is physically inconsistent; hence, it is fixed to the minimum physical value, i.e., 0 nm/μN.

Methods based on two CRMs, i.e., the ISO 14577-2 method M4 and the ODR single-step method, are less sensitive to the force range. In fact, all estimated parameters are statistically compatible, as shown in Figure 4. Methods based on two CRMs are generally highly accurate on FS (with an absolute relative accuracy smaller than 1%), and slightly worse on W, as shown in Figure 7. In particular, the ODR single-step method, due to its definition, provides significantly more accurate estimates also on W, with benefits in terms of the relative uncertainty, which is halved when resorting to the ODR rather than the M4, i.e., from 50% to 25%.

A slight overestimation of the frame compliance is obtained when a larger range of forces is considered for W (W7), i.e., when smaller forces are also considered for W (1mN and 2.5 mN). For the mechanical characterization of W, this overestimation leads to a trend (for M4) or a bias (for ODR) likely due to the superposition of the strong non-linearity of the *C_f_*, which cannot be characterized by the considered methods, and due to the incipient indentation size effect of W.

In summary, the best calibration results suggest limiting the applied force range to larger forces on W, while increasing the number of force levels on FS is, in general, beneficial in terms of both relative accuracy and relative uncertainty.

## 5. Conclusions

This work highlighted the criticalities of the current calibration framework for area shape function and frame compliance in nanoindentation based on indirect calibration methods.

The work showed that calibration methods based on the use of a single sample are less accurate and more uncertain than methods that require the use of two certified reference materials. In particular, methods based on the use of a single sample provide a relative accuracy that is worse by one order of magnitude and a relative uncertainty that is at least double. Furthermore, this work highlighted a strong dependency of methods based on the use of one sample on the considered force range. Lastly, the standard version of such approaches is autocalibration and although practical, it yields poor performance in terms of relative accuracy and uncertainty.

Summarizing the results from the work, a practical suggestion for the calibration of the area shape function and frame compliance can be outlined within an uncertainty evaluation framework as follows:

- Calibration methods based on two certified reference materials, e.g., ISO 14577-2 method 4, optimize the accuracy and uncertainty of characterization.
- Multiple calibration indentation datasets are needed to avoid the severe underestimation of calibration uncertainty.
- A cost-effective approach to cater for the effect of the calibration indentation dataset can be obtained by the bootstrap simulation of such datasets.
- Indentation on tungsten certified reference material shall be greater than 5 mN to avoid characterization bias.
- The single-step method, based on orthogonal distance regression, further improves the accuracy and uncertainty of the ISO 14577-2 method 4.

Future work will compare the performance of calibration methods on other materials with industrial relevance, such as Ge, aluminum alloys, and titanium alloys, provided that their mechanical characteristics have been calibrated using an independent calibration approach, e.g., the pulse–echo method.

## Figures and Tables

**Table 1:** ISO 14577-2 calibration methods. Notes provide procedural suggestions, including application scope and certified reference materials (CRMs) to be used.

Method #	*A_p_* Calibration	*C_f_* Calibration	Input Required	Notes
1	Direct by AFM	Indirect	*A_p_* calibration	CRM: W
2	Indirect	Indirect	None	CRM: Al, or SiO_2_
3	Direct by AFM	Indirect	*A_p_* calibration, calibrated *E_r_*	CRM: W
4	Indirect	Indirect	Calibrated *E_r,1_*, *E_r,2_*	CRM 1 (*A_p_*): SiO_2_; CRM 2 (*C_f_*): W
5	Indirect	Indirect	Calibrated *E_r,_*_1_, *E_r,_*_2_, elastic deformation	For spherical indenters
6	Indirect	Indirect	None	For micro- and macro-range (CRM shall not exhibit indentation size effect, nor significant pile-up and sink-in)

**Table 2:** CRM properties and tested maximum forces. Intervals of calibrated CRM properties are confidence intervals at a 95% confidence level (k = 2). *H_IT_* was evaluated on historical data.

Certified Reference Material	Calibrated Property	Tested Maximum Forces/mN
SiO_2_ (FS)	E = (73.0 ± 0.5) GPa	0.5, 1, 2.5, 5, 7.5, 10, 15, 20
	υ = (0.163 ± 0.002)	
	H_IT_ = (8.5 ± 0.5) GPa	
W	E = (414.3 ± 5.6) GPa	1, 2.5, 5, 7.5, 10, 15, 20
	υ = (0.279 ± 0.005)	
	H_IT_ = (7.0 ± 0.5) GPa	

**Table 3:** Selected calibration methods. *I* is the number of considered force levels.

Short Name	Applied Method	CRM	Used Force Range/mN	*I*
M2-FS5	ISO 14577-2 M2 with use of calibrated *E_r_*	SiO_2_	[1,2,3,4,5,6,7,8,9,10]	5
M2-FS8			[0.5–20]	8
M2AUTOCAL-FS5	ISO 14577-2 M2		[1,2,3,4,5,6,7,8,9,10]	5
M2AUTOCAL-FS8			[0.5–20]	8
M4-FS5-W5	ISO 14577-2 M4	SiO_2_, W	SiO_2_: [1,2,3,4,5,6,7,8,9,10] W: [5,6,7,8,9,10,11,12,13,14,15,16,17,18,19,20]	5
M4-FS8-W5			SiO_2_: [0.5–20] W: [5,6,7,8,9,10,11,12,13,14,15,16,17,18,19,20]	*A_p_*: 8 *C_f_*: 5
M4-FS8-W7			SiO_2_: [0.5–20] W: [1,2,3,4,5,6,7,8,9,10,11,12,13,14,15,16,17,18,19,20]	*A_p_*: 8 *C_f_*: 7
ODR-FS5-W5	ODR Single-step method	SiO_2_, W	SiO_2_: [1,2,3,4,5,6,7,8,9,10] W: [5,6,7,8,9,10,11,12,13,14,15,16,17,18,19,20]	5
ODR-FS8-W5			SiO_2_: [0.5–20] W: [5,6,7,8,9,10,11,12,13,14,15,16,17,18,19,20]	*A_p_*: 8 *C_f_*: 5
ODR-FS8-W7			SiO_2_: [0.5–20] W: [1,2,3,4,5,6,7,8,9,10,11,12,13,14,15,16,17,18,19,20]	*A_p_*: 8 *C_f_*: 7

**Table 4:** *p*-values and relative contribution to sum of squares (SS) due to the indentation dataset variability, which in this work has been obtained by bootstrapping. A relevant contribution is highlighted for *p*-values smaller than 5% (0.05). Notice that the area shape function parameters are most sensitive to the indentation dataset.

	$C_{f}$		$a_{0}$		$a_{1}$		$a_{2}$	
Method	*p*-Value	$SS_{B}/SS_{TOT}$	*p*-Value	$SS_{B}/SS_{TOT}$	*p*-Value	$SS_{B}/SS_{TOT}$	*p*-Value	$SS_{B}/SS_{TOT}$
M2-FS5	<0.001	95.7%	<0.001	80.2%	<0.001	87.6%	<0.001	97.0%
M2-FS8	>0.99	<1‰		25.8%		32.8%		43.1%
M2AUTOCAL-FS5	<0.001	95.3%		73.2%		83.0%		93.1%
M2AUTOCAL-FS8	<0.001	68.2%		36.8%		58.6%		95.8%
M4-FS5-W5	>0.99	1.9%		39.5%		43.2%		50.7%
M4-FS8-W5	>0.99	1.8%		27.7%		35.8%		53.7%
M4-FS8-W7	<0.001	6.8%		33.8%		44.4%		70.5%
ODR-FS5-W5	<0.001	38.0%		21.3%		21.0%		19.9%
ODR-FS8-W5	<0.001	47.7%		22.9%		25.6%		36.2%
ODR-FS8-W7	<0.001	40.1%		19.4%		21.6%		30.6%

**Figure A1:**
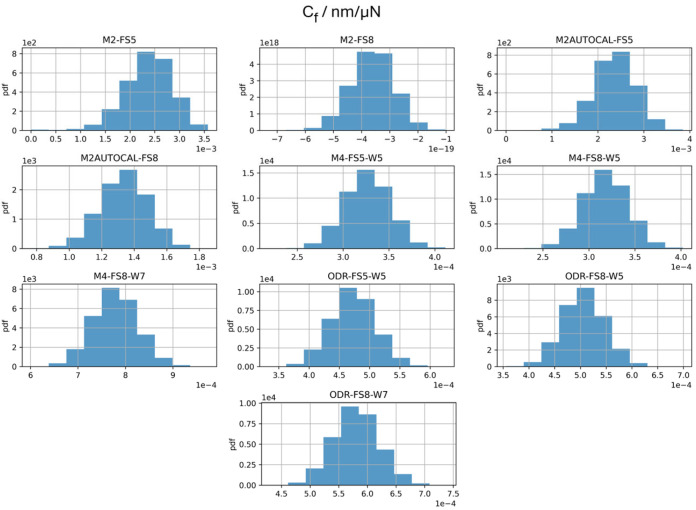
Empirical probability density function (pdf) of the 10,000 calibrated parameters for the 10 calibration methods for the frame compliance (*C_f_*).

**Figure A2:**
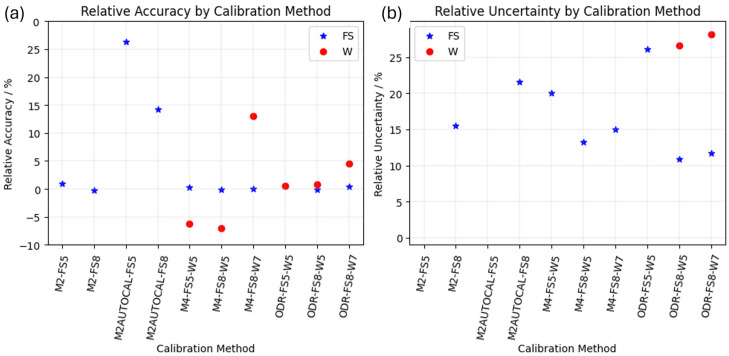
(**a**) Relative accuracy and (**b**) relative uncertainty for the *E_IT_* of SiO_2_ (FS blue star) and W (red circle). Not all values are represented to improve visualization (extreme values are excluded).

## Data Availability

The data used in this study are openly available on Zenodo at 10.5281/zenodo.17054480. The code for generating bootstrap samples is openly available on Zenodo at 10.5281/zenodo.17054597.

## Abbreviations

The following abbreviations are used in this manuscript:

CRM	Certified Reference Material
FS	Fused Silica (SiO2)
IIT	Instrumented Indentation Test
ISO	International Organization for Standardization
